# *cis*-Aqua­dichlorido[pyrimidin-2(1*H*)-one-κ*N* ^3^]copper(II)

**DOI:** 10.1107/S1600536808018771

**Published:** 2008-06-28

**Authors:** Mukhtar A. Kurawa, Christopher J. Adams, A. Guy Orpen

**Affiliations:** a School of Chemistry, University of Bristol, Bristol BS8 1TS, England

## Abstract

In the title compound, [CuCl_2_(C_4_H_4_N_2_O)(H_2_O)], the Cu^II^ cation is coordinated by two chloride anions, one pyrimidin-2-one N atom and one water mol­ecule, giving a slightly distorted square-planar geometry. In the crystal structure, the pyrimidin-2-one rings stack along the *b* axis, with an inter­planar distance of 3.306 Å, as do the copper coordination planes (inter­planar spacing = 2.998 Å). The coordination around the Jahn–Teller-distorted Cu^II^ ion is completed by long Cu⋯O [3.014 (5) Å] and Cu⋯Cl [3.0194 (15) Å] inter­actions with adjacent mol­ecules involved in this stacking. Several N—H⋯Cl, O—H⋯Cl and O—H⋯O inter­molecular hydrogen bonds form a polar three-dimensional network.

## Related literature

A similar coordination environment and geometry about the copper atom was described by Crass *et al.* (1996 ▶) for [Cu(C_12_H_18_N_4_)Cl_2_(H_2_O)_2_].
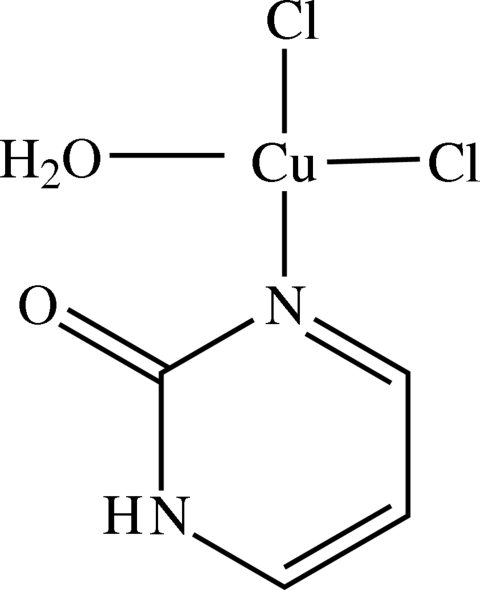


## Experimental

### 

#### Crystal data

[CuCl_2_(C_4_H_4_N_2_O)(H_2_O)]
*M* *_r_* = 248.53
Monoclinic, 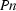

*a* = 9.6104 (4) Å
*b* = 3.7942 (2) Å
*c* = 10.7375 (4) Å
β = 107.991 (4)°
*V* = 372.39 (3) Å^3^
*Z* = 2
Mo *K*α radiation
μ = 3.59 mm^−1^
*T* = 100 (2) K
0.28 × 0.08 × 0.06 mm

#### Data collection

Oxford Diffraction Gemini-R Ultra diffractometer
Absorption correction: multi-scan (*CrysAlis RED*; Oxford Diffraction, 2007 ▶) *T*_min_ = 0.739, *T*_max_ = 0.810
6373 measured reflections
1866 independent reflections
1462 reflections with *I* > 2σ(*I*)
*R*_int_ = 0.046

#### Refinement

*R*[*F* ^2^ > 2σ(*F* ^2^)] = 0.033
*wR*(*F* ^2^) = 0.098
*S* = 1.01
1866 reflections
106 parameters
4 restraints
H atoms treated by a mixture of independent and constrained refinement
Δρ_max_ = 0.73 e Å^−3^
Δρ_min_ = −0.67 e Å^−3^
Absolute structure: Flack (1983 ▶), 765 Friedel pairs
Flack parameter: 0.03 (2)

### 

Data collection: *CrysAlis CCD* (Oxford Diffraction, 2007 ▶); cell refinement: *CrysAlis RED* (Oxford Diffraction, 2007 ▶); data reduction: *CrysAlis RED*; program(s) used to solve structure: *SHELXS97* (Sheldrick, 2008 ▶); program(s) used to refine structure: *SHELXL97* (Sheldrick, 2008 ▶); molecular graphics: *SHELXTL* (Sheldrick, 2008 ▶); software used to prepare material for publication: *SHELXTL*.

## Supplementary Material

Crystal structure: contains datablocks I, global. DOI: 10.1107/S1600536808018771/fj2126sup1.cif

Structure factors: contains datablocks I. DOI: 10.1107/S1600536808018771/fj2126Isup2.hkl

Additional supplementary materials:  crystallographic information; 3D view; checkCIF report

## Figures and Tables

**Table 1:** Selected bond lengths (Å)

Cu1—O2	1.976 (4)
Cu1—N1	2.040 (4)
Cu1—Cl1	2.2440 (14)
Cu1—Cl2	2.2466 (14)

**Table 2:** Hydrogen-bond geometry (Å, °)

*D*—H⋯*A*	*D*—H	H⋯*A*	*D*⋯*A*	*D*—H⋯*A*
N2—H2*B*⋯Cl1^i^	0.86	2.51	3.333 (5)	160
O2—H2⋯Cl2^ii^	0.85 (2)	2.52 (4)	3.279 (4)	149 (7)
O2—H1⋯O1^iii^	0.84 (2)	1.86 (4)	2.629 (6)	152 (7)

Symmetry codes: (i) 
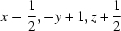
; (ii) 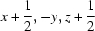; (iii) 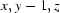.

**Fig. 1.**
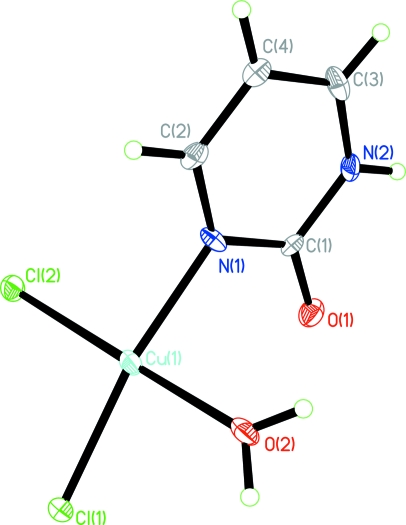
The molecular stucture of I with atom labels and 50% probability displacement ellipsoids for non-H atoms.

**Fig. 2.**
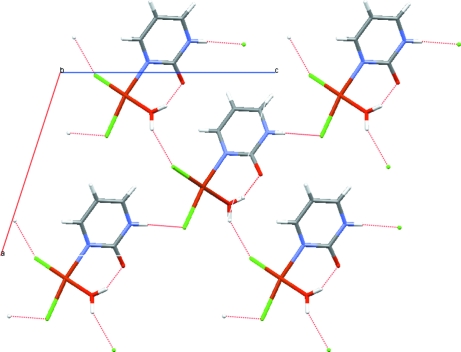
Packing of I viewed down the b axis showing the polar packing with various N—H···Cl, O—H···Cl and O—H···O intermolecular hydrogen bonds.

**Fig. 3.**
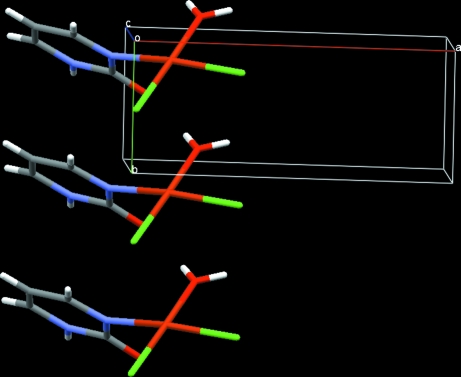
π—π stacking of I viewed along the b axis.

## supplementary crystallographic information

### Comment

Mechanochemistry is a technique currently attracting increasing interest, in part because of its potential to offer an environmentally friendly and sustainable means for solid state synthesis. We sought to broaden the scope of this still relatively under-utilized technique by reacting CuCl_2_.2H_2_O and 2-hydroxypyrimidine hydrochloride under mechanochemical conditions to synthesize [C_4_H_5_N_2_O]_2_[CuCl_4_]. However, the title compound I was obtained instead, and crystal structure determination at 100 (2) K revealed a square planar molecule in the polar space group *Pn*. Crass *et al.* (1996) reported the structure of a related compound [Cu(C_12_H_18_N_4_)Cl_2_(H_2_O)_2_] with a similar type of coordination environment at copper but a different hydrogen bonding network.

### Experimental

CuCl_2_.2H_2_O and 2-hydroxypyrimidine hydrochloride in a 1:2 molar ratio were ground in an agate mortar. The resulting powder was dissolved in acetonitrile and the solution left to evaporate slowly at room temperature. Green, needle-like crystals of the title compound were obtained after a few days.

### Refinement

H atoms bonded to O atom were located in the difference map and refined with distance restraints of O—H = 0.84 (2) Å with *U*_iso_(H) = 1.2*U*_eq_(O). Other H atoms were positioned geometrically and refined using a riding model, with C—H = 0.93 Å and N—H = 0.86 Å, with *U*_iso_(H) = 1.2 times *U*_eq_(C, N).

### Crystal data

**Table:**

[CuCl_2_(C_4_H_4_N_2_O)(H_2_O)]	*F*_000_ = 246
*M**_r_* = 248.53	*D*_x_ = 2.217 Mg m^−^^3^
Monoclinic, *P**n*	Mo *K*α radiation λ = 0.71073 Å
Hall symbol: P -2yac	Cell parameters from 2806 reflections
*a* = 9.6104 (4) Å	θ = 2.2–30.0º
*b* = 3.7942 (2) Å	µ = 3.59 mm^−^^1^
*c* = 10.7375 (4) Å	*T* = 100 (2) K
β = 107.991 (4)º	Needle, green
*V* = 372.39 (3) Å^3^	0.28 × 0.08 × 0.06 mm
*Z* = 2	

### Data collection

**Table:**

Oxford Diffraction Gemini-R Ultra diffractometer	1866 independent reflections
Radiation source: fine-focus sealed tube	1462 reflections with *I* > 2σ(*I*)
Monochromator: graphite	*R*_int_ = 0.046
*T* = 100(2) K	θ_max_ = 30.1º
1° wide ω scans	θ_min_ = 2.5º
Absorption correction: multi-scan(CrysAlis RED; Oxford Diffraction, 2007)	*h* = −13→13
*T*_min_ = 0.739, *T*_max_ = 0.810	*k* = −5→5
6373 measured reflections	*l* = −12→15

### Refinement

**Table:**

Refinement on *F*^2^	Hydrogen site location: inferred from neighbouring sites
Least-squares matrix: full	H atoms treated by a mixture of independent and constrained refinement
*R*[*F*^2^ > 2σ(*F*^2^)] = 0.033	*w* = 1/[σ^2^(*F*_o_^2^) + (0.0567*P*)^2^] where *P* = (*F*_o_^2^ + 2*F*_c_^2^)/3
*wR*(*F*^2^) = 0.098	(Δ/σ)_max_ < 0.001
*S* = 1.01	Δρ_max_ = 0.73 e Å^−^^3^
1866 reflections	Δρ_min_ = −0.67 e Å^−^^3^
106 parameters	Extinction correction: none
4 restraints	Absolute structure: Flack (1983), 767 Friedel pairs
Primary atom site location: structure-invariant direct methods	Flack parameter: 0.03 (2)
Secondary atom site location: difference Fourier map	

### Special details

**Table:**

Experimental. CrysAlis RED, Oxford Diffraction Ltd., Version 1.171.32.5 Empirical absorption correction using spherical harmonics, implemented in SCALE3 ABSPACK scaling algorithm.
Geometry. All e.s.d.'s (except the e.s.d. in the dihedral angle between two l.s. planes) are estimated using the full covariance matrix. The cell e.s.d.'s are taken into account individually in the estimation of e.s.d.'s in distances, angles and torsion angles; correlations between e.s.d.'s in cell parameters are only used when they are defined by crystal symmetry. An approximate (isotropic) treatment of cell e.s.d.'s is used for estimating e.s.d.'s involving l.s. planes.
Refinement. Refinement of *F*^2^ against ALL reflections. The weighted *R*-factor *wR* and goodness of fit *S* are based on *F*^2^, conventional *R*-factors *R* are based on *F*, with *F* set to zero for negative *F*^2^. The threshold expression of *F*^2^ > σ(*F*^2^) is used only for calculating *R*-factors(gt) *etc*. and is not relevant to the choice of reflections for refinement. *R*-factors based on *F*^2^ are statistically about twice as large as those based on *F*, and *R*- factors based on ALL data will be even larger.

### Fractional atomic coordinates and isotropic or equivalent isotropic displacement parameters (Å^2^)

**Table:**

	*x*	*y*	*z*	*U*_iso_*/*U*_eq_	
Cu1	0.12489 (4)	0.17338 (18)	0.33630 (4)	0.01410 (18)	
Cl1	0.34639 (14)	0.2487 (4)	0.31142 (14)	0.0148 (3)	
Cl2	0.01546 (13)	0.5320 (4)	0.16807 (13)	0.0144 (3)	
N1	−0.0593 (5)	0.1582 (12)	0.3924 (5)	0.0113 (10)	
N2	−0.1732 (5)	0.2409 (12)	0.5539 (4)	0.0141 (9)	
H2B	−0.1698	0.3200	0.6298	0.017*	
O1	0.0558 (4)	0.4563 (11)	0.5852 (4)	0.0168 (8)	
O2	0.2175 (4)	−0.1468 (12)	0.4838 (4)	0.0200 (9)	
H2	0.308 (3)	−0.20 (2)	0.511 (7)	0.024*	
H1	0.183 (7)	−0.239 (18)	0.539 (5)	0.024*	
C1	−0.0520 (6)	0.2951 (15)	0.5126 (5)	0.0120 (11)	
C2	−0.1804 (5)	0.0019 (14)	0.3222 (6)	0.0128 (11)	
H2A	−0.1849	−0.0799	0.2394	0.015*	
C3	−0.2949 (6)	0.0750 (15)	0.4845 (6)	0.0157 (12)	
H3A	−0.3728	0.0450	0.5177	0.019*	
C4	−0.3019 (6)	−0.0484 (15)	0.3644 (5)	0.0147 (11)	
H4A	−0.3850	−0.1628	0.3120	0.018*	

### Atomic displacement parameters (Å^2^)

**Table:**

	*U*^11^	*U*^22^	*U*^33^	*U*^12^	*U*^13^	*U*^23^
Cu1	0.0126 (3)	0.0189 (3)	0.0130 (3)	0.0033 (3)	0.0072 (2)	0.0032 (3)
Cl1	0.0113 (6)	0.0206 (7)	0.0134 (7)	0.0007 (5)	0.0054 (5)	0.0018 (5)
Cl2	0.0147 (5)	0.0155 (7)	0.0137 (6)	0.0025 (5)	0.0053 (5)	0.0039 (6)
N1	0.011 (2)	0.009 (2)	0.017 (2)	−0.0004 (17)	0.0093 (19)	−0.0011 (19)
N2	0.017 (2)	0.019 (2)	0.007 (2)	−0.0019 (18)	0.0043 (17)	−0.0007 (18)
O1	0.0177 (18)	0.017 (2)	0.0130 (18)	−0.0032 (16)	0.0012 (15)	0.0008 (16)
O2	0.0140 (19)	0.028 (3)	0.022 (2)	0.0025 (18)	0.0117 (17)	0.0089 (19)
C1	0.010 (2)	0.015 (3)	0.009 (2)	0.001 (2)	−0.0006 (19)	0.002 (2)
C2	0.013 (2)	0.011 (3)	0.012 (2)	0.006 (2)	0.001 (2)	0.001 (2)
C3	0.023 (3)	0.012 (3)	0.019 (3)	0.004 (2)	0.014 (2)	0.003 (2)
C4	0.015 (3)	0.014 (3)	0.013 (3)	0.002 (2)	0.001 (2)	0.000 (2)

### Geometric parameters (Å, °)

**Table:**

Cu1—O2	1.976 (4)	O1—C1	1.247 (7)
Cu1—N1	2.040 (4)	O2—H2	0.85 (5)
Cu1—Cl1	2.2440 (14)	O2—H1	0.84 (5)
Cu1—Cl2	2.2466 (14)	C2—C4	1.390 (8)
N1—C2	1.316 (7)	C2—H2A	0.9300
N1—C1	1.372 (7)	C3—C4	1.354 (8)
N2—C3	1.335 (7)	C3—H3A	0.9300
N2—C1	1.384 (7)	C4—H4A	0.9300
N2—H2B	0.8600		
			
O2—Cu1—N1	87.87 (17)	H2—O2—H1	104 (7)
O2—Cu1—Cl1	87.99 (12)	O1—C1—N1	124.4 (5)
N1—Cu1—Cl1	168.71 (13)	O1—C1—N2	119.4 (5)
O2—Cu1—Cl2	178.89 (13)	N1—C1—N2	116.2 (5)
N1—Cu1—Cl2	91.22 (14)	N1—C2—C4	124.0 (5)
Cl1—Cu1—Cl2	93.03 (5)	N1—C2—H2A	118.0
C2—N1—C1	119.3 (5)	C4—C2—H2A	118.0
C2—N1—Cu1	122.5 (4)	N2—C3—C4	118.1 (5)
C1—N1—Cu1	118.0 (4)	N2—C3—H3A	120.9
C3—N2—C1	124.8 (5)	C4—C3—H3A	120.9
C3—N2—H2B	117.6	C3—C4—C2	117.6 (5)
C1—N2—H2B	117.6	C3—C4—H4A	121.2
Cu1—O2—H2	125 (5)	C2—C4—H4A	121.2
Cu1—O2—H1	130 (5)		
			
O2—Cu1—N1—C2	−111.0 (4)	Cu1—N1—C1—N2	−172.3 (4)
Cl1—Cu1—N1—C2	−179.5 (5)	C3—N2—C1—O1	179.5 (5)
Cl2—Cu1—N1—C2	68.4 (4)	C3—N2—C1—N1	−1.5 (8)
O2—Cu1—N1—C1	64.2 (4)	C1—N1—C2—C4	−2.8 (8)
Cl1—Cu1—N1—C1	−4.3 (10)	Cu1—N1—C2—C4	172.3 (4)
Cl2—Cu1—N1—C1	−116.4 (4)	C1—N2—C3—C4	−0.5 (8)
C2—N1—C1—O1	−178.0 (5)	N2—C3—C4—C2	0.9 (8)
Cu1—N1—C1—O1	6.6 (7)	N1—C2—C4—C3	0.7 (8)
C2—N1—C1—N2	3.1 (7)		

### Hydrogen-bond geometry (Å, °)

**Table:**

*D*—H···*A*	*D*—H	H···*A*	*D*···*A*	*D*—H···*A*
N2—H2B···Cl1^i^	0.86	2.51	3.333 (5)	160
O2—H2···Cl2^ii^	0.85 (2)	2.52 (4)	3.279 (4)	149 (7)
O2—H1···O1^iii^	0.84 (2)	1.86 (4)	2.629 (6)	152 (7)

Symmetry codes: (i) *x*−1/2, −*y*+1, *z*+1/2; (ii) *x*+1/2, −*y*, *z*+1/2; (iii) *x*, *y*−1, *z*.
